# Validating predictive models on EHRs data : lessons learnt from the NCDR-IMPACT-score applied on a French cardiac catheterization cohort

**DOI:** 10.1186/s12872-026-06065-4

**Published:** 2026-06-27

**Authors:** S Quennelle, S Malekzadeh-Milani, N Garcelon, A Burgun, D Bonnet, A Neuraz

**Affiliations:** 1 PaRis Artificial Intelligence Research InstitutE (PR[AI]RIE), ANR-19-P3IA-0001, Paris, France; 2 Clinical Bioinformatics Group, Imagine Institute, Université Paris Cité, INSERM UMR 1163, Paris, France; 3 M3C-Necker, Hôpital Universitaire Necker-Enfants malades, Assistance Publique-Hôpitaux de Paris, Paris, France; 4 LadHyX, CNRS, Ecole Polytechnique, Institut Polytechnique de Paris, Palaiseau, France; 5 Imagine Institute, Data Science Platform, INSERM UMR 1163, Université de Paris, Paris, France

**Keywords:** Risk adjustment, Congenital heart disease, Generalizability, Artificial intelligence, Machine learning

## Abstract

**Objective:**

We present a natural language processing pipeline to extract and re-compute a pre-existing score, the IMPACT-score, in a children’s hospital belonging to the APHP hospitals.

**Materials and methods:**

Predictive variables and adverse events occurrences were extracted from the EHR of each patient. Data extraction involved rule-based and machine learning approaches depending on the format of the data. The machine learning text-classifiers were trained on active learning annotated dataset. Once the registry was automatically populated, we computed the IMPACT-score model in our patients and we performed a logistic regression analysis to find the specific odd ratio fitting our cohort and obtain the IMPACT-score-Necker.

**Results:**

We extracted clinical data from 2,980 patients. When applied to our hospital cohort, the IMPACT-score-Necker achieved an AUC of 0.719 whereas the original IMPACT-score achieved an AUC of 0.642. As a reminder, the IMPACT-score achieved an AUC of 0.752 in the NCDR-IMPACT validation cohort.

**Discussion:**

Local calibration of the IMPACT-score on our cohort enhanced predictive accuracy, improving the AUC from 0.642 to 0.719, and addressing differences between our population and the original NCDR-IMPACT cohort. This reinforces the need for model adaptation to local data, as patient demographic and clinical variations can significantly impact performance. Local EHR data warehouses could be leveraged for recalibration and continuous monitoring, ensuring that AI tools remain accurate, ethical, and practical for clinicians in their clinical practice.

**Conclusion:**

Our results underscore the need for real-world model validation, and EHRs offer a reliable source for training and validating AI models.

**Supplementary Information:**

The online version contains supplementary material available at https://doi.org/10.1186/s12872-026-06065-4.

## Background and significance

Advances in interventional techniques have opened the field of cardiac catheterization (CC) in congenital heart diseases (CHD) for diagnosis or to treat heart defects. CC does not come without risks. Short-term complications include damage to blood vessels, allergic reactions to the contrast dye, and in rare cases, more serious events such as heart attack, stroke, or arrhythmias [1–3]. Therefore, CC requires a meticulous evaluation of the patient’s medical history, a comprehensive assessment of potential contraindications, and a thorough consideration of alternative methods.

International and national cardiology societies recommend the calculation of risk scores to identify patients at risk of adverse events (AEs) related to CC [4, 5]. Jayaram et al. [6] developed a logistic regression model trained on a large cohort of 39,725 procedures from the U.S. National Cardiovascular Data Registry Improving Pediatric and Adult Congenital Treatment (NCDR-IMPACT) data collection [7]. The model achieved good discrimination, with an AUC of 0.76 and 0.75 in the derivation and validation cohorts, respectively. This model is considered a standardized risk score for CC. However, its calculation has not been generalized. The first question raised by such a low adoption, is whether the models have to be validated locally before being implemented. Recent studies show that algorithms performing well in controlled settings often fail to generalize to real-world populations, and a score developed for one specific population may lack generalizability and transferability to other patient groups [8–11]. An example among many is the Framingham Risk Score. Based on a population cohort from Framingham, Massachusetts, it leads to over- or underestimate risks of developing cardiovascular diseases in non-white populations [12, 13]. The second remark is that a prediction model is calculated at a given period of time and may become obsolete when medical techniques and practices evolve. Finally, a classic risk score involves assigning odds ratios to risk factors. The risk will be calculated by adding the odds ratios for each patient based on the number of risk factors found for that patient. However, scores cannot take into account all relevant factors, especially those related to the context, or incidental events like the operator’s fatigue.

Developing a new, population-specific, risk score is not always feasible since it requires a large database and a resource-intensive data collection to build the training data set. Re-use of routine care data may help for overcoming this obstacle, with Electronic Health Records (EHRs) being used to populate registries and fine-tune predictive models.

## Objective

Our objective was to evaluate the IMPACT predictive model on the cohort of CHD pediatric patients that underwent catheterization at Necker-Enfants malades hospital (NEMH), and to compare its performance to a model trained locally. The study is based on retrospective EHR data to extract all variables.

## Materials and methods

### Patient selection

We considered all patients hospitalized at the tertiary pediatric cardiology center of the NEMH, a children’s hospital belonging to the AP-HP group and located in Paris. We included all patients who underwent CCs between July 2010 and December 2016. In case of multiple procedures, only the first CC was considered. The study was approved by the APHP ethical committee and registered under #2022 0503160104. The data underlying this article cannot be shared publicly due to its highly sensitive and identifiable nature.

### Data extraction

We followed the IMPACT-score protocol, which includes 17 input variables: age and weight, hemodynamic measurements (left ventricular end-diastolic pressure, pulmonary vascular resistance, pulmonary to systemic flow ratio, mean pulmonary artery pressure, systolic pulmonary artery pressure, mixed venous saturation, systemic saturation) used to define four levels of hemodynamic vulnerability, lab tests (platelet count, fibrinogen, prothrombin time, partial thromboplastin time) used to define coagulation disorders, comorbidities (renal insufficiency, single ventricle, anticoagulant therapy) and the type of procedure categorized in six levels of risk (appendix A). The list of AEs was the initial list established by the NCDR-IMPACT. Structured data (age, weight, hemodynamic measurements and lab tests) and unambiguous clinical terms (e.g., single ventricle) were automatically extracted using straightforward rule-based methods [14]. Other data, including signs and symptoms, comorbidities, procedure type and AEs, were extracted using a deep learning classifier based on text embedding and a recurrent neural network. This deep learning classifier was trained on a dataset annotated using an active learning algorithm. The extraction method has been published previously [15] and evaluated on a subset of patients [14]. We made the same assumption as the NCDR-IMPACT protocol that the information required for the IMPACT-score calculation, if positive, was reported in the EHR and missing values were imputed using non-pathological values. 

### IMPACT-score calculation and recalibration

We computed two different IMPACT-score models:

1. The initial IMPACT model : we applied the odd ratios from the original IMPACT-score study to the data collected at our site. The predictions made by the model were stored for all patients from our cohort.
2. A predictive model was trained on the NEMH cohort using logistic regression. The cohort was split, with 70% used for derivation and 30% for validation. Due to the small number of patients in extreme risk and vulnerability categories, which resulted in unstable parameter estimates and inflated confidence intervals, adjustments were made to ensure sufficient events per parameter and preserve model stability. Risk categories were grouped into a single variable with six levels, and vulnerability factors into one variable with four levels, instead of treating them as distinct categories. This recalibrated model is referred to as the IMPACT-score-Necker throughout the article.

We compared the performances of these two models to predict AEs.

## Results

### Cohort description

A total of 2,980 patients underwent CC during the study period (Table 1). All had an EHR and were included in the cohort. The type of procedure was detected for 2,610 (88%) patients. The most frequent procedure was CC for diagnosis. A total of 211 (7.1%) patients had experienced AEs, corresponding exactly to the event rate observed in the original IMPACT-score derivation cohort.

**Table 1:** Baseline characteristics of patients

	*n*	Mean (SD)
Overall	2,980	
Weight (kg)	2,536	18.8 (17.8)
Age (years)	2,977	4.9 (5.7)
LVEDP (mmHg)	161	13.9 (7.0)
PVR^a^ (mmHg)	140	7.4 (6.6)
QPQS^a^ (mmHg)	136	2.8 (1.8)
mPAP (mmHg)	1,494	27.2 (18.9)
sPAP (mmHg)	1,414	39.9 (26.0)
SvO2 (%)	503	70.3 (11.8)
SAT^a^ (%)	1,868	94.7 (7.1)
Platelet count (G/L)	840	280.1 (98.3)
Fibrinogen (g/L)	75	2.6 (1.2)
PT	549	87.1 (13.1)
PTT	326	1.0 (0.2)

	n	%
Renal failure	23	0.8
Single-ventricle physiology	353	11.8
Anticoagulation therapy	565	19.0
Coagulation disorder	32	1.1
Procedure type	2,610	87.6
AEs	211	7.1

*LVEDP* left ventricular end-diastolic pressure, *PVR* pulmonary vascular resistance, *QPQS* pulmonary to systemic flow ratio, *mPAP* mean pulmonary artery pressure, *sPAP* systolic pulmonary artery pressure, *SVO2* mixed venous saturation, *SAT* systemic saturation, *PT* prothrombin time, *PTT* partial thromboplastin time

^a^PVR, QPQS and SAT were obtained using the structured data when available in the catheterization report and through regular expressions applied to the hospitalization report

### Patients’ scores

**Fig. 1:**
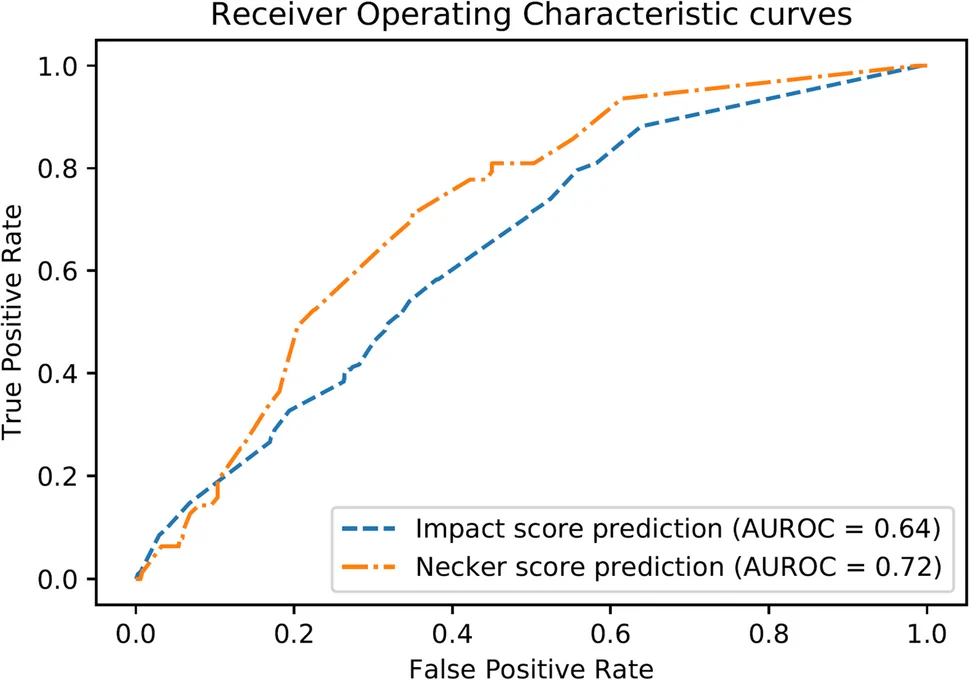
Receiver operating characteristic curves for the IMPACT score and the IMPACT-Necker score in our local cardiac catheterization cohort

When applied to our hospital validation cohort, the IMPACT-Score-Necker achieved an AUC of 0.719 whereas the original IMPACT-score achieved an AUC of 0.642 (Fig. 1; Table 2). As a reminder, the IMPACT-score achieved an AUC of 0.752 in the initial NCDR-IMPACT validation cohort.

**Table 2:** Predictors of AEs, comparison between the IMPACT-score and the IMPACT-score necker odds ratio

Predictors	IMPACT-score-Necker Odds Ratio (95% CI)	IMPACT-score Predictor	IMPACT-score Reduced Model Odds Ratio (95% CI)
Constant	0,03 (0.02, 0.04)	Risk category 1	reference
Risk Category ^a^	1,24 (1.14, 1.35)	Risk category 2	1.88 (1.49, 2.38)
		Risk category 3	3.89 (3.06, 4.96)
		Risk category 4	3.87 (3.03, 4.93)
		Risk category 5	8.91 (6.95, 11.43)
		Risk category 6	12.80 (9.83, 16.68)
Markers of Hemodynamic Vulnerability	1,31 (1.11, 1.55)	0 Markers of Hemodynamic Vulnerability	reference
		1 Markers of Hemodynamic Vulnerability	1.21 (1.00, 1.46)
		2 Markers of Hemodynamic Vulnerability	1.73 (1.44, 2.07)
		> 2 Markers of Hemodynamic Vulnerability	2.45 (2.05, 2.93)
Renal Insufficiency	2.40 (0.69, 8.30)	Renal Insufficiency	2.16 (1.69, 2.76)
Single Ventricle	2,42 (1.71, 3.42)	Single Ventricle	1.23 (1.10, 1.38)
Coagulation Disorder	3,46 (1.38, 8.65)	Coagulation Disorder	2.42 (1.78, 3.27)

Coagulation disorders and high-risk procedures (e.g., risk category 6) were the major risk factors and contributed to a significantly elevated risk score. In patients with a low-risk score (2.5) who experienced adverse events, some complications were highly unpredictable, such as ketamine-induced laryngeal spasm, pulmonary embolism (which is very rare in children), or operator mishandling. CI indicates confidence interval

^a^Risk categories were defined by the IMPACT-score protocole [6]

## Discussion

### Adoption of models trained on different populations

Our study illustrates that local score calibration results in better predictions. This result was obtained even if the initial IMPACT-score was based on an a-priori large and representative population. By retraining the IMPACT-score on our local cohort, we managed to enhance the model’s precision. The AUC improved from 0.642 when using the original IMPACT coefficients to 0.719 when the IMPACT-score was recalibrated on our cohort. This could potentially be attributed to differences between the patients in the NCDR-IMPACT Registry and those from NEMH. The NCDR-IMPACT Registry gathered 39,725 procedures from 74 US centers while our cohort comprises 2,980 procedures from NEMH. Patients from the IMPACT-Registry were older, the mean age was 9.7 (± 12.7) years compared to 4.9 (± 5.7) years in the NEMH’s cohort. Specifically, the proportion of newborns is different in the data sets: only 6.2% of the patients were less than 30 days old in the NCDR-IMPACT Registry compared to 12% of patients in the NEMH’s cohort. Additionally, single ventricle physiology was more prevalent in the NCDR-IMPACT Registry patients (20%) compared to NEMH’s cohort (12%). This finding illustrates a well-known issue: some algorithms, trained on a given population dataset, cannot generalize to a different population.

This question has been raised by several authors: Kennedy et al. [16] showed that the calibration of statistical models on a specific population can improve risk prediction performances; Shah et al. [17] claimed that transparent reporting of local performance of the model as well as consequences for local patient subpopulations was necessary for safe and ethical AI; Adams et al. [18], highlighted that AI models may include implicit and/or explicit bias, notably for individuals from under-represented groups. What we call bias in statistics and “dataset shift” or “domain shift” in ML [19, 20], occurs when a model underperforms due to a mismatch between the data used for model development and the data on which it is applied [21–23]. The commonly accepted assumption is that models trained on a diverse and large enough dataset (in our case, a national registry) should generalize to other sites and patients without requiring new training. Numerous examples [24, 25] have shown that this assumption is false, and as a promising solution, domain adaptation has attracted considerable attention in recent years [20].

External validation of ML algorithms (i.e. the verification of the algorithm performance on a dataset derived from a source that is different from the training set) is often considered the ultimate test to conclusively judge the safety, reliability and generalizability of a model [26]. Specifically in the case of rare diseases where datasets are hard to obtain, models rarely go beyond standard validation and real-world studies are exceptionally performed [27]. Our findings illustrate that even relatively simple models, such as logistic regression, may require domain adaptation before being applied to a new population. This underscores the importance of local data warehouses and robust data extraction pipelines to properly evaluate both AI and non-AI algorithms on local patient cohorts prior to implementation in routine clinical practice.

### Clinical adoption of AI models

EHR data reuse looks like the best solution for validating models, and recalibrating scores. Rationale for re-using EHR data is twofold: (i) it provides data at lower costs, (ii) it provides data that are similar to those used in real-world clinical implementation. The development of the IMPACT-score was made possible by the extensive NCDR-IMPACT Registry in the US, the largest clinical registry of patients undergoing interventional catheterization procedures. The increasing adoption of data warehouses may enable practitioners to enhance risk prediction by recalibrating an existing model, assuming that efficient clinical data extraction tools are available on site. Moreover, new EHR data will accumulate over time, allowing for continuous performance monitoring and regular model updating [28].

In clinical cardiovascular practice, risk prediction tools are recommended to assist with treatment, diagnostic, and follow-up [8]. However, practitioners are often reluctant to calculate risk scores due to time-consuming data collection. We implemented a pipeline that automatically computes patients’ scores without requiring additional data entry. Once validated, adoption by clinicians would be easier if the system is interoperable with EHRs, as demonstrated in the clinical study of the TREWS machine learning system for sepsis [29].

Our study echoes the recommendations made by the Ethics Guidelines for Trustworthy AI [30] and by the American Medical Association. Beyond evaluation and interoperability with EHRs, responsible AI, validated in real-world settings is mandatory before translating AI outputs into actionable decisions that clinicians routinely use. We believe that local validation and fine-tuning of the algorithms should be not only mandatory steps but also incentives toward their practical application in clinical decision-making.

### Limitation

This study has several limitations. First, the comparison involved two risk scores evaluated in different populations. Although the retrained version demonstrated improved performance in our cohort, this comparison should be interpreted cautiously. Importantly, data collection methodologies differed substantially between settings. In the original NCDR-IMPACT registry, data were collected prospectively by trained personnel using the Pediatric and Congenital Cardiac Code nomenclature and a standardized set of data elements and definitions, under rigorous quality assurance procedures. In contrast, our data were obtained through automated extraction from the EHR. Differences in data acquisition processes may therefore have introduced measurement bias and could partially explain discrepancies in model performance. Nevertheless, while a comprehensive replication of the original data collection framework would have been ideal, such an approach was not feasible within our institutional setting. The development and maintenance of large prospective registries require substantial financial and human resources. For predictive models to achieve meaningful clinical impact, their external validation and local implementation must remain feasible across institutions at a lower cost. In this context, automated data extraction represents a pragmatic and scalable strategy for external validation of risk scores in real-world settings.

## Conclusion

We replicated the NCDR-IMPACT model and recalculated the IMPACT score on the EHRs from Necker tertiary pediatric cardiology center. The model trained on local data achieved a better AUC (0.719) than the original IMPACT-score (0.642). Our experience illustrates that retraining statistical models on target populations can enhance model performance.

## Supplementary Information

Supplementary Material 1: Reproduction of the Final Procedure-Type Risk Categories of the IMPACT-score [6]. List of adverse events related to cardiac catheterization. Discrimination (AUROC, AUPRC) and Operating Characteristics of the two scores.

## Data Availability

Due to the highly sensitive and identifying nature of health data, our patient data will not be shared. The code of the model used to extract the data and calculate the logistic regression will be made available.

## Abbreviations

AEs: Adverse Events
AI: Artificial Intelligence
AP-HP: Assistance Publique Hôpitaux de Pars
AUC: Area under the ROC Curve
CC: Cardiac Catheterization
CHD: Congenital Heart Disease
EHRs: Electronic Health Records
LLM: Large Language Models
ML: Machine Learning
NCDR-IMPACT: National Cardiovascular Data Registry Improving Pediatric and Adult Congenital Treatment
NEMH: Necker-Enfants Malades Hospital
NLP: Natural Language Processing
