# Supplementary material for: Validating predictive models on EHRs data : lessons learnt from the NCDR-IMPACT-score applied on a French cardiac catheterization cohort
Source: BMC Cardiovasc Disord. 2026 Jun 27;26:887. doi: 10.1186/s12872-026-06065-4 (PMC13647702; doi:10.1186/s12872-026-06065-4)
Supplement: Supplementary file 1 [file 12872_2026_6065_MOESM1_ESM.docx]

Supplementary appendices

**Appendix A. Reproduction of the Final Procedure-Type Risk Categories of the IMPACT-score** [[6]](https://www.zotero.org/google-docs/?YNpFfS)

|  | **Risk Category 1** | **Risk Category 2** | **Risk Category 3** | **Risk Category 4** | **Risk Category 5** | **Risk Category 6** |
| --- | --- | --- | --- | --- | --- | --- |
| **Diagnostic Case** |  | Diagnostic Cath (age >1 year) |  | Diagnostic Cath (age 30 days-1year) |  | Diagnostic Cath (age <30 days) |
| **Valvuloplasty** |  | Pulmonic Valvuloplasty (age >30 days) |  | Aortic Valvuloplasty (age >30 days) | Aortic Valvuloplasty (age <30 days)  Pulmonic Valvuloplasty (age <30 days) |  |
| **Device or coil closure** |  | ASD/PFO  coronary artery fistula  PDA (weight >6kg)  Fontan fenestration or baffle leak | Systemic artery (excluding aorta) or systemic vein  Pulmonary or systemic AVM  Perivalvar leak | PDA closure (weight 3–6kg)  Systemic artery to pulmonary artery connection  Systemic vein to pulmonary vein connection | VSD  PDA (weight <3kg) |  |
| **Balloon Angioplasty** |  | Conduit | Angioplasty for Coarctation  Proximal or distal PA  Fontan baffle or fenestration | Caval or non-caval vein  Atrial septum s/p atrial switch | Atrial septum  Dilation of Sano  Pulmonary Vein  Systemic Artery (other than aorta)  Systemic to pulmonary artery shunt |  |
| **Stent Placement** |  | Conduit | Stent Placement for Coarctation  Proximal or distal PA  Fontan baffle or fenestration | Caval or non-caval vein  Atrial septum s/p atrial switch | Atrial septum  PDA  Stenting of Sano  Pulmonary Vein  Systemic Artery (other than aorta)  Systemic to pulmonary artery shunt |  |
| **Stent Redilatation** |  | Stent re-dilation (other than for coarctation) | Stent re-dilation for coarctation |  |  |  |
| **Other** | Myocardial biopsy s/p heart transplantation | Elective pericardiocentesis | Transcatheter pulmonary valve placement  Myocardial Biopsy (excluding s/p heart transplant) | Snare Foreign Body  Balloon valvotomy of mitral valve | Balloon atrial septostomy  Perforation of atretic pulmonary valve  Atrial septal perforation |  |

*Abbreviations: PDA, patent ductus arteriosus; VSD, ventricular septal defect; kg, kilograms; PA, pulmonary artery; AVM, arteriovenous malformation; PFO, patent foramen ovale; ASD, atrial septal defect*

**Appendix B. List of adverse events related to cardiac catheterization**

Cardiac arrest

Arrhythmia requiring an antiarrhythmic medication, cardioversion, or temporary or permanent pacemaker

New onset of valvular regurgitation

Cardiac tamponade (requiring pericardial drainage)

Air embolism resulting in clinical change

Embolic stroke less than 72 hours after the cardiac catheterization

Device malposition or thrombus (requiring surgery)

Device embolization (requiring device retrieval)

New requirement for dialysis

Event requiring intubation

Event requiring extracorporeal membrane oxygenation

Event requiring left ventricular assist device

Significant bleeding event less than 72 hours after the cardiac catheterization (defined as bleeding associated with a drop in hemoglobin of ≥3 g/dl, bleeding requiring transfusion or an intervention or surgery at the source of the bleeding)

Other vascular complications requiring treatment

Unplanned cardiac, vascular, or other surgery (because of catheterization complications)

Subsequent cardiac catheterization (because of catheterization complications)

Death before hospital discharge

**Appendix C : Discrimination (AUROC, AUPRC) and Operating Characteristics of the two scores**

|  | **AUROC** | **AUPRC** | **specificity** | **sensibility** | **precision** | **recall** |
| --- | --- | --- | --- | --- | --- | --- |
| IMPACT-score | 0.72 | 0.12 | 0.65 | 0.71 | 0.10 | 0.93 |
| IMPACT-score-Necker | 0.64 | 0.11 | 0.72 | 0.42 | 0.07 | 1.0 |

With a complication prevalence of 7%, the model achieved an AUPRC of 0.12, representing a modest improvement over the baseline event rate and indicating limited positive predictive enrichment despite acceptable discrimination.
